# All-Trans Retinoic Acid Nanocrystals for Enhanced Anticancer Activity Against Neuroblastoma Cancer Stem Cells

**DOI:** 10.3390/pharmaceutics18101219

**Published:** 2026-09-26

**Authors:** Divya Ajmeera, Rajanna Ajumeera

**Affiliations:** Cell Biology Department, ICMR-National Institute of Nutrition, Hyderabad 500007, Telangana, India; arajanna@gmail.com

**Keywords:** all-trans retinoic acid, ATRA nanocrystals, nanoformulation, neuroblastoma, cancer stem cells, CD133^+^ cells, apoptosis

## Abstract

**Background/Objectives:** Neuroblastoma is a high-risk pediatric malignancy in which cancer stem cells (CSCs) contribute to treatment resistance and disease recurrence. All-trans retinoic acid (ATRA) has established differentiation-inducing activity in neuroblastoma but is constrained by poor aqueous solubility and formulation challenges. **Methods:** ATRA nanocrystals (ATRA-NCs) stabilized with Poloxamer 407 were developed and characterized for their physicochemical properties. Their anticancer activity was evaluated in IMR-32 neuroblastoma cells and CD133+ CSCs, with unformulated ATRA used as the comparator, using multiple in vitro biological assays. **Results:** ATRA-NCs exhibited a mean hydrodynamic diameter of 194 ± 5.3 nm and a zeta potential of −60.1 mV. Compared with unformulated ATRA, ATRA-NCs produced greater concentration-dependent cytotoxicity and enhanced apoptotic responses in IMR-32 neuroblastoma cells. In CD133+ CSCs, ATRA-NCs reduced cell viability and suppressed the expression of stemness-associated genes, including SOX2, NANOG, LGR5, OCT4, BMI-1, and ALDH1A1. The formulation also reduced colony-forming capacity, impaired spheroid formation, and inhibited cellular migration. **Conclusions:** These findings demonstrate enhanced in vitro anticancer activity against neuroblastoma cancer cells and CD133+ CSCs following ATRA nanocrystallization and indicate modulation of multiple functional phenotypes associated with neuroblastoma CSCs. ATRA nanocrystallization may therefore provide a formulation approach for enhancing ATRA activity against neuroblastoma and warrants further preclinical evaluation..

## 1. Introduction

Neuroblastoma is among the most aggressive pediatric solid tumors and remains a major cause of cancer-related mortality in children [1]. Despite advances in multimodal treatment, including chemotherapy, radiotherapy, surgery, and immunotherapy, patients with high-risk disease continue to experience treatment resistance and recurrence [2]. Increasing evidence identifies cancer stem cells (CSCs) as important contributors to tumor initiation, progression, therapeutic resistance, and disease recurrence in neuroblastoma [2]. Consequently, therapeutic strategies that modulate CSC-associated properties may provide a complementary approach for improving the management of neuroblastoma [2].

All-trans retinoic acid (ATRA), a naturally occurring metabolite of vitamin A, is an established differentiation-inducing agent with documented activity in neuroblastoma [3,4]. ATRA promotes neuronal differentiation and growth arrest in neuroblastoma cells and has also been reported to influence stemness-associated characteristics [4,5]. However, its pharmaceutical application is constrained by poor aqueous solubility and formulation-related limitations, which can affect its dissolution and effective pharmaceutical delivery [6]. These characteristics have stimulated interest in formulation strategies capable of improving the pharmaceutical performance of ATRA and potentially enhancing its biological activity.

Several nanoformulation approaches have been investigated to address the delivery limitations of ATRA. Polymeric systems have been developed to improve ATRA solubilization and release. In particular, polymeric micellar formulations based on modified polyvinyl alcohol have demonstrated enhanced aqueous solubilization of ATRA and increased cytotoxicity compared with free ATRA in neuroblastoma models [7]. Similarly, ATRA-loaded anionic liposomes have been evaluated in SK-N-SH human neuroblastoma cells and compared with free ATRA, with the liposomal formulation producing an enhanced differentiation response [8]. These studies support the potential of nanoformulation strategies to modify the pharmaceutical and biological performance of ATRA. However, these approaches rely on polymeric or lipid-based carrier systems for incorporating the drug.

Drug nanocrystals represent a distinct formulation approach for improving the pharmaceutical performance of poorly water-soluble drugs. Nanocrystals consist predominantly of the active pharmaceutical ingredient in nanoscale form and are generally stabilized by a relatively small amount of surfactant or polymer. Reduction of drug particle size increases the surface area available for interaction with the dissolution medium and can enhance dissolution kinetics and, for some poorly soluble drugs, apparent saturation solubility [9,10]. Such improvements in dissolution may contribute to enhanced drug exposure and bioavailability, depending on the physicochemical characteristics of the drug, formulation, and route of administration [9,10]. The high proportion of active pharmaceutical ingredients and the absence of a substantial drug-encapsulating carrier matrix distinguish nanocrystals from conventional polymeric and lipid-based nanoparticles [9]. These characteristics provide a rationale for investigating nanocrystallization as a formulation strategy for improving the pharmaceutical performance of poorly water-soluble ATRA.

Within the broader field of nanomedicine, nanoscale drug-delivery systems have been investigated for their potential to improve drug distribution within solid tumors. The enhanced permeability and retention (EPR) effect has been proposed as one mechanism contributing to the passive accumulation of appropriately sized nanomaterials in tumor tissue, owing to abnormal tumor vasculature and impaired lymphatic drainage [11]. However, the magnitude of the EPR effect varies considerably among tumor types and biological settings, and current evidence indicates that EPR alone does not fully explain nanoparticle delivery to solid tumors [11]. Thus, tumor-selective accumulation should be considered a potential advantage of appropriately designed nanoscale delivery systems rather than a universal property of nanomedicines. Direct assessment of tumor accumulation and biodistribution is required to establish whether such effects occur with a specific formulation.

Despite the established differentiation-inducing activity of ATRA in neuroblastoma and the growing application of nanoformulation strategies to improve its pharmaceutical performance, the use of ATRA nanocrystals for investigating neuroblastoma CSC-associated phenotypes remains comparatively less explored. This distinction is important because responses observed in bulk neuroblastoma cells may not fully reflect the effects of ATRA on CSC-associated functional properties. Previous studies have demonstrated that retinoic acid can influence stemness-associated characteristics in neuroblastoma [5]. However, an integrated evaluation of ATRA nanocrystals in a defined CD133^+^ neuroblastoma CSC population, together with assessment of multiple functional CSC-associated endpoints, has not been comprehensively described. Such an approach provides an opportunity to determine whether nanoscale formulation of ATRA can enhance its biological activity relative to the unformulated drug.

Accordingly, the present study developed ATRA nanocrystals (ATRA-NCs) stabilized with Poloxamer 407 and characterized their physicochemical properties and in vitro biological activity. The formulation was evaluated in IMR-32 neuroblastoma cells and in the CD133^+^ CSC population isolated from the same model. ATRA-NCs were compared with unformulated ATRA using multiple biological endpoints, including cell viability, apoptosis, cell-cycle distribution, stemness-associated gene expression, colony formation, spheroid formation, and cellular migration. The study was designed to determine whether nanocrystal formulation could enhance the in vitro anticancer activity of ATRA and modulate functional phenotypes associated with neuroblastoma CSCs.

## 2. Materials and Methods

### 2.1. Materials

All-trans retinoic acid (ATRA; purity ≥98%) and Poloxamer 407 were procured from Sigma-Aldrich (St. Louis, MO, USA). Analytical-grade methanol, chloroform, and acetonitrile (HPLC grade) were purchased from Merck (Darmstadt, Germany). Cell-culture media, including RPMI-1640 and DMEM, fetal bovine serum (FBS), penicillin–streptomycin, and trypsin–EDTA, were obtained from Thermo Fisher Scientific (Waltham, MA, USA). Reagents used for in vitro biological assays included MTT (3-(4,5-dimethylthiazol-2-yl)-2,5-diphenyltetrazolium bromide; Sigma-Aldrich), Annexin V–FITC/PI apoptosis detection kit (BD Biosciences, San Jose, CA, USA), Hoechst 33342 (Thermo Fisher Scientific), JC-1 mitochondrial membrane potential assay kit (Cayman Chemical, USA), and DCFH-DA (2′,7′-dichlorodihydrofluorescein diacetate; Sigma-Aldrich).

Human neuroblastoma IMR-32 cells were obtained from the National Centre for Cell Science (NCCS, Pune, India). Cells were maintained under standard culture conditions at 37 °C in a humidified atmosphere containing 5% CO_2_ and cultured in the appropriate medium supplemented with 10% FBS and 1% penicillin–streptomycin.

Throughout the manuscript, “ATRA” refers to unformulated all-trans retinoic acid (Drug), whereas “ATRA-NCs” refers to the ATRA nanocrystal formulation (Formulation).

### 2.2. Preparation and Characterization of ATRA-NCs

#### 2.2.1. Nanocrystal Preparation

ATRA nanocrystals were prepared using a modified antisolvent precipitation method followed by high-pressure homogenization [12]. ATRA (10 mg/mL) was dissolved in methanol:chloroform (1:1, *v*/*v*) and slowly injected into deionized water containing 1% Poloxamer 407 under magnetic stirring at 1000 rpm for 30 min. The resulting suspension was subjected to high-pressure homogenization for 20 cycles at 15,000 psi using a Nano DeBee homogenizer (BEE International, South Easton, MA, USA). Residual organic solvents were removed by rotary evaporation using a Buchi R-210 system (Switzerland).

The resulting nanosuspension was lyophilized using a Labconco FreeZone system (USA) at −50 °C for 48 h in the presence of 5% mannitol as a cryoprotectant to obtain the ATRA-NC powder.

#### 2.2.2. Morphological Characterization

The surface morphology and particle shape of ATRA-NCs were evaluated by scanning electron microscopy (SEM; Carl Zeiss Sigma 300 VP, Carl Zeiss Microscopy GmbH, Jena, Germany) and transmission electron microscopy (TEM; FEI Tecnai G2 F20, FEI Company, Hillsboro, OR, USA) [13]. For SEM analysis, samples were sputter-coated with gold (~10 nm) and examined at an accelerating voltage of 10 kV. For TEM analysis, samples were deposited onto copper grids, air-dried, and examined at an accelerating voltage of 200 kV.

#### 2.2.3. Colloidal Characterization

The mean hydrodynamic particle size, polydispersity index (PDI), and zeta potential of ATRA-NCs were determined by dynamic light scattering (DLS) using a Zetasizer Nano ZS90 (Malvern Instruments, Worcestershire, UK) [14]. ATRA-NCs were dispersed in ultrapure water at a concentration of 1 mg/mL and sonicated for 5 min before analysis. Measurements were performed in triplicate. The hydrodynamic diameter and PDI were determined from the particle-size distribution, and the zeta potential was measured under the same dispersion conditions.

#### 2.2.4. Structural and Solid-State Characterization

Fourier Transform Infrared Spectroscopy (FTIR): ATRA-NC powder (2 mg) was mixed with KBr (200 mg), compressed into pellets, and analyzed over the wavenumber range of 4000–650 cm^−1^ at a resolution of 4 cm^−1^ using an Agilent Cary 630 FTIR spectrometer [15].

Powder X-Ray Diffraction (PXRD): PXRD analysis was performed using a Bruker D8 Advance diffractometer operated at 40 kV and 30 mA with Cu-Kα radiation (λ = 1.5406 Å). Diffraction patterns were recorded over a 2θ range of 5–60° at a scanning rate of 2°/min.

Differential Scanning Calorimetry (DSC): DSC analysis was performed using a PerkinElmer DSC 6000. ATRA-NC samples (2–5 mg) were weighed into aluminum pans, sealed, and analyzed over a temperature range of 50–300 °C at a heating rate of 10 °C/min under a nitrogen atmosphere [15]. These analyses ensure comprehensive information on particle morphology, colloidal stability, chemical integrity, crystallinity, and thermal behavior, supporting reproducible formulation and biological evaluation.

### 2.3. In Vitro Biological Assays

#### 2.3.1. Cell Culture and Maintenance

Human neuroblastoma IMR-32 cells were maintained in RPMI-1640 medium supplemented with 10% FBS and 100 IU/mL penicillin–streptomycin at 37 °C in a humidified atmosphere containing 5% CO_2_.

#### 2.3.2. CSC Isolation and Characterization

CD133^+^ cancer stem cells were isolated from IMR-32 cells using magnetic-activated cell sorting (MACS; Miltenyi Biotec, Bergisch Gladbach, Germany). Cells were labeled with CD133 microbeads according to the manufacturer’s instructions and separated using MACS columns. The isolated CD133^+^ population was further verified by immunofluorescence microscopy.

#### 2.3.3. Nuclear Fragmentation and Morphological Changes

To assess nuclear morphology and apoptotic changes, IMR-32 cells were treated with ATRA or ATRA-NCs at concentrations of 10, 20, and 30 µg/mL. Following treatment, cells were collected, washed twice with phosphate-buffered saline (PBS), and fixed with methanol:acetic acid (3:1, *v*/*v*). Cells were subsequently stained with 4′,6-diamidino-2-phenylindole (DAPI) for 30 min at room temperature. After staining, cells were mounted using glycerol:PBS (1:1, *v*/*v*) and examined under a fluorescence microscope for nuclear condensation, fragmentation, and apoptotic bodies [16].

#### 2.3.4. Cell Viability (MTT Assay)

Cell viability was evaluated using the MTT assay [17]. IMR-32 cells were seeded at 4000 cells/well in 96-well plates and treated with ATRA or ATRA-NCs at concentrations ranging from 0 to 100 µg/mL for 24–96 h. Following treatment, 20 µL of MTT solution (5 mg/mL) was added to each well and incubated for 4 h. The resulting formazan crystals were dissolved in 200 µL DMSO, and absorbance was measured at 570 nm using a Bio-Rad iMark microplate reader. Cell viability was calculated using the following equation:

Cell viability (%) = [(OD of treated cells − OD of blank)/(OD of control − OD of blank)] × 100.(1)

#### 2.3.5. Apoptosis (Annexin V–FITC/PI)

Apoptotic and necrotic cell populations were quantified using Annexin V–FITC/PI staining followed by flow cytometry [18]. IMR-32 cells (1 × 10^5^ cells) were treated with ATRA or ATRA-NCs for 48 h, washed with PBS, and stained according to the manufacturer’s instructions. Stained cells were analyzed using a BD FACSCalibur flow cytometer, and viable, early apoptotic, late apoptotic, and necrotic populations were quantified.

#### 2.3.6. Cell-Cycle Analysis

For cell-cycle analysis, cells were fixed in 70% ethanol overnight, treated with RNase A (100 µg/mL), and stained with propidium iodide (PI; 50 µg/mL). DNA-content profiles were acquired by flow cytometry, and the proportions of cells in the G0/G1, S, and G2/M phases were determined using ModFit LT software [19].

#### 2.3.7. Colony Formation Assay

IMR-32 cells were treated with ATRA or ATRA-NCs at concentrations of 10, 20, and 30 µg/mL for 48 h. Following treatment, cells were trypsinized and seeded into 6-well plates at a density of 50–100 cells/well. Cells were cultured for 11–15 days, with medium replacement every 3 days. Colonies were fixed with 4% paraformaldehyde for 30 min and stained with 0.5% crystal violet for 30 min. Colonies were photographed using a digital camera and counted manually. Colony-forming activity was expressed relative to the untreated control [20].

#### 2.3.8. Spheroid Formation Assay

Three-dimensional spheroids were generated by culturing IMR-32 cells in ultra-low-attachment 96-well plates at a density of 100–300 cells/well in serum-free RPMI-1640 supplemented with 20 ng/mL human recombinant epidermal growth factor (hrEGF), 20 ng/mL human recombinant basic fibroblast growth factor (hrbFGF), 1% B27 supplement, and 1× penicillin–streptomycin. Following 7 days of incubation to allow spheroid formation, cells were treated with ATRA or ATRA-NCs at concentrations of 1, 5, 10, 20, and 30 µg/mL for 72 h. Spheroid morphology and size were assessed by phase-contrast microscopy, and mean spheroid diameter was determined using ImageJ software [21].

#### 2.3.9. Cell Migration Assay (Scratch Wound-Healing Assay)

IMR-32 cells were cultured in 6-well plates and allowed to reach confluence. A scratch wound was created on confluent IMR-32 monolayers using a sterile pipette tip and treated with ATRA or ATRA-NCs at concentrations of 10–30 µg/mL. Migration was monitored at 0 and 48 h after scratching using an inverted microscope. The wound area was quantified using ImageJ software, and the percentage of wound closure was calculated as follows [22]:

Wound closure (%) = [(Initial wound area − Final wound area)/Initial wound area] × 100.(2)

#### 2.3.10. Gene Expression Analysis by Quantitative RT-PCR

IMR-32 cells were treated with ATRA (Drug) or ATRA-NCs (Formulation) at concentrations of 5–30 µg/mL for 24 h, alongside an untreated control group. Total RNA was extracted using TRIzol reagent (Invitrogen, Carlsbad, CA, USA), and complementary DNA (cDNA) was synthesized using the RevertAid First Strand cDNA Synthesis Kit (Thermo Fisher Scientific, USA) according to the manufacturer’s protocol. Quantitative real-time PCR (qRT-PCR) was performed using SYBR Green PCR Master Mix on a StepOne Real-Time PCR System (Applied Biosystems, Waltham, MA, USA). Relative mRNA expression levels of the stemness-associated genes SOX2, NANOG, LGR5, OCT4, BMI-1, and ALDH1A1 were analyzed. GAPDH was used as the internal control, and relative expression was calculated using the 2^−ΔΔCt^ method [23]. Expression data were normalized to the untreated control, which was assigned a relative expression value of 1.0.

### 2.4. Statistical Analysis

All experiments were performed in triplicate (*n* = 3). Data are expressed as mean ± SD. Statistical significance was assessed using one-way analysis of variance (ANOVA) followed by Tukey’s post hoc test in GraphPad Prism version 9. A *p*-value < 0.05 was considered statistically significant.

## 3. Results and Discussion

### 3.1. Preparation of ATRA-NCs

ATRA nanocrystals (ATRA-NCs) were successfully prepared by modified antisolvent precipitation followed by high-pressure homogenization and subsequent lyophilization. The resulting formulation was obtained as a dry powder suitable for physicochemical and biological evaluation.

Nanocrystallization is an established strategy for improving the pharmaceutical performance of poorly water-soluble drugs. Reduction of particle size increases the surface area available for interaction with the dissolution medium and can thereby enhance dissolution kinetics. The nanoscale state may also influence apparent solubility and, depending on the physicochemical characteristics of the drug and formulation, may contribute to improved drug dissolution [10,24]. These physicochemical advantages provide the rationale for investigating ATRA in nanocrystalline form.

In the present study, successful formation of ATRA-NCs was evaluated through complementary morphological, colloidal, spectroscopic, diffraction, and thermal analyses. These physicochemical characteristics were subsequently examined in relation to the biological responses observed in IMR-32 neuroblastoma cells and CD133^+^ CSCs.

### 3.2. Physicochemical Characterization of ATRA-NCs

#### 3.2.1. Morphological Characterization

SEM and TEM were used to compare the morphology and particle dimensions of plain ATRA and ATRA-NCs (Figure 1). Plain ATRA exhibited relatively large, irregular crystalline structures, whereas ATRA-NCs showed markedly reduced particle dimensions with nanoscale morphology. The high-magnification SEM image showed individual ATRA-NC particle dimensions within approximately 101–199 nm, while TEM images further supported the nanoscale nature of the formulated particles.

The observed reduction in particle dimensions is consistent with the combined use of antisolvent precipitation and high-pressure homogenization, established approaches for producing drug nanocrystals [10]. Reduction in particle size increases the available surface area of poorly soluble drug particles and can contribute to enhanced dissolution kinetics [10,24]. The particle dimensions observed by electron microscopy were broadly consistent with the hydrodynamic size obtained by DLS. Differences between these measurements are expected because electron microscopy provides dimensions of prepared/dried particles, whereas DLS measures the hydrodynamic diameter of particles dispersed in liquid and can be influenced by the surrounding medium and surface-associated stabilizer [14].

#### 3.2.2. Colloidal Properties

Particle Size and Polydispersity: DLS analysis showed a Z-average hydrodynamic diameter of 194 nm for ATRA-NCs, with a PDI of 0.832 (Figure 2A). The size distribution further indicated a D50 of 213.8 nm and D90 of 870.7 nm, demonstrating a relatively broad particle-size distribution. PDI is an important descriptor of particle-size distribution and should be considered when evaluating dispersion uniformity and formulation reproducibility [14].

The relatively high PDI observed in the present formulation indicates that the preparation was not highly monodisperse. The presence of a larger particle fraction, reflected by the D90 value, should therefore be considered during future formulation optimization and scale-up. Nevertheless, the formulation demonstrated the intended nanoscale particle characteristics and was suitable for the subsequent in vitro evaluations.

Zeta Potential: The zeta potential of ATRA-NCs was −60.1 mV (Figure 2B), indicating a strongly negative surface potential. Zeta potential provides information regarding the electrostatic characteristics of dispersed particles and contributes to the understanding of particle–particle interactions and colloidal behavior [14,25]. Poloxamer 407, a non-ionic stabilizer used in the formulation, can additionally provide steric stabilization at the particle–aqueous interface.

The combination of surface charge and steric stabilization provides a plausible physicochemical basis for maintaining the dispersed state of the nanocrystals. However, cellular interaction with nanoscale particles is influenced by multiple factors, including particle size, surface composition, protein adsorption, and cell-surface characteristics [25]. The present study therefore establishes the physicochemical surface characteristics of ATRA-NCs, whereas the specific molecular mechanisms governing their cellular interaction were not directly investigated.

#### 3.2.3. Structural and Solid-State Characterization

FTIR Analysis: FTIR spectra of plain ATRA and ATRA-NCs showed the characteristic vibrational features associated with the principal functional groups of ATRA (Figure 3A). Plain ATRA exhibited characteristic bands at 3048.96, 2931.55, 1677.30, and 1600.89 cm^−1^, corresponding predominantly to =C–H, aliphatic C–H, carboxylic C=O, and C=C stretching vibrations, respectively, consistent with previously reported FTIR characteristics of ATRA [6]. These major characteristic bands were retained in ATRA-NCs, indicating preservation of the principal chemical functionalities of ATRA following formulation. Minor differences in the relative intensities of some bands may reflect changes in the local molecular environment following nanocrystal formation and association with formulation components [6].

Overall, the FTIR profile supports successful formulation of ATRA without substantial alteration of its characteristic chemical functionalities and complements the PXRD and DSC findings.

PXRD Analysis: The PXRD profiles of Poloxamer 407, mannitol, their physical mixture, plain ATRA, and the ATRA formulation are presented in Figure 3B. Plain ATRA exhibited distinct diffraction reflections characteristic of a crystalline material. The ATRA formulation also retained discernible diffraction features, together with reflections attributable to the formulation components.

The persistence of diffraction features indicates that ATRA was not converted completely into an amorphous state during formulation. Differences in the relative intensity and appearance of reflections may reflect changes in the crystalline characteristics of the drug and contributions from the formulation components. PXRD comparison with the individual components and physical mixtures is therefore important for interpreting the solid-state characteristics of the final formulation [13].

Taken together, the PXRD profile supports the presence of crystalline material within the ATRA formulation and indicates that the solid-state characteristics of the formulation differ from those of plain ATRA.

DSC Analysis: DSC thermograms of plain ATRA, mannitol, Poloxamer 407, ATRA-NCs, and the corresponding physical mixtures are shown in Figure 3C. Plain ATRA exhibited a characteristic endothermic transition with a peak temperature of approximately 185.21 °C, while mannitol and Poloxamer 407 exhibited thermal transitions at approximately 167.68 °C and 56.41 °C, respectively.

The ATRA-NC formulation displayed a modified thermal profile compared with the individual components, including a prominent low-temperature endothermic transition at approximately 49.81 °C and a broader thermal event extending into the higher-temperature region. Because the formulation contains mannitol and Poloxamer 407, the thermal transitions observed in the formulation cannot be attributed exclusively to ATRA. Comparison with the individual components and physical mixtures therefore provides the appropriate basis for interpretation [26].

The altered thermal profile, together with the PXRD findings, demonstrates modification of the solid-state characteristics of the formulation following processing. However, the data do not support describing ATRA-NCs as completely amorphous.

### 3.3. In Vitro Biological Evaluation

#### 3.3.1. Identification and Characterization of CD133^+^ Neuroblastoma CSCs

CD133^+^ CSCs were identified and enriched from the IMR-32 neuroblastoma cell population using MACS and subsequently characterized by immunofluorescence microscopy (Figure 4). The CD133^−^ population showed DAPI-stained nuclei with minimal or absent CD133-associated fluorescence, whereas the CD133^+^ population displayed clear CD133-associated fluorescence. The corresponding merged images further demonstrated the presence of CD133-associated signal in the CD133^+^ population.

CD133 is a commonly investigated surface marker for enrichment of stem-like populations in neuroblastoma, and CD133^+^ neuroblastoma cells have been associated with CSC-related characteristics [27,28]. The CD133^+^ population therefore provided a relevant CSC-enriched model for subsequent evaluation of ATRA and ATRA-NCs.

Because CD133 expression alone does not define all functional characteristics of a CSC population, complementary functional and molecular endpoints—including colony formation, spheroid formation, migration, and stemness-associated gene expression—were subsequently evaluated. This integrated approach provides a broader assessment of CSC-associated phenotypes than reliance on CD133 expression alone.

#### 3.3.2. Nuclear Morphological Changes Following ATRA and ATRA-NC Treatment

DAPI staining was used to evaluate nuclear morphological changes in IMR-32 neuroblastoma cells following treatment with ATRA or ATRA-NCs at 10, 20, and 30 µg/mL (Figure 5A). Untreated cells predominantly exhibited intact nuclei with relatively uniform staining, whereas treated cells showed increasing nuclear condensation and fragmentation with increasing treatment concentration.

Quantitative analysis demonstrated an increase in the proportion of apoptotic nuclei following treatment, with ATRA-NCs producing a greater response than ATRA at corresponding concentrations (Figure 5B). Nuclear condensation and fragmentation are characteristic morphological features associated with apoptotic cell death [29]. These findings therefore provide morphological support for the apoptotic response observed following ATRA and ATRA-NC treatment.

#### 3.3.3. Cell Viability (MTT Assay)

##### The Cytotoxic Effects of ATRA and ATRA-NCs

The cytotoxic effects of ATRA and ATRA-NCs were evaluated in IMR-32 neuroblastoma cells using the MTT assay. As shown in Figure 6A, both treatments produced a concentration-dependent reduction in cell viability across the concentration range of 1–100 µg/mL, with ATRA-NCs producing a significantly greater reduction at corresponding concentrations. Time-dependent evaluation at the respective IC_50_ concentrations further demonstrated a progressive decline in cell viability over 6–96 h (Figure 6B). The IC_50_ value of ATRA-NCs was lower than that of unformulated ATRA, indicating greater in vitro cytotoxic activity of the nanocrystal formulation.

The enhanced response may be related to the physicochemical advantages associated with nanoscale formulation, including improved dispersion and increased surface area available for dissolution [10,24]. However, the present study did not directly measure cellular uptake or intracellular drug retention; therefore, these factors should be regarded as possible contributors rather than established mechanisms.

#### 3.3.4. Apoptotic and Necrotic Responses

Annexin V–FITC/PI staining was performed to further characterize treatment-associated cell-death responses in IMR-32 neuroblastoma cells and CSCs (Table 1; Figure 7). Compared with untreated cells, ATRA and ATRA-NC treatment increased the apoptotic and necrotic cell fractions, with a more pronounced response observed following ATRA-NC treatment.

Quantitative analysis demonstrated increased total apoptotic populations following treatment, supporting the DAPI observations of nuclear condensation and fragmentation. The greater apoptotic response following ATRA-NC treatment is consistent with the enhanced cytotoxic activity observed in the MTT assay.

These findings indicate that the reduction in cell viability following ATRA-NC treatment was accompanied by increased apoptotic cell death. Because Annexin V/PI staining identifies cell-death states rather than the upstream molecular pathway responsible for apoptosis, the present results support an enhanced apoptotic response without establishing a specific intracellular apoptotic mechanism.

#### 3.3.5. Cell-Cycle Distribution

Flow-cytometric analysis demonstrated a shift in cell-cycle distribution following treatment with ATRA and ATRA-NCs (Table 2; Figure 8). In IMR-32 cancer cells, the G0/G1 population decreased from 33.6 ± 0.9% in untreated cells to 25.4 ± 0.7% following ATRA treatment, with a corresponding increase in the S-phase population from 66.4 ± 1.1% to 74.6 ± 1.3% (*p* < 0.01). In the IMR-32 CSC population, the G0/G1 fraction decreased from 36.4 ± 0.8% in untreated cells to 25.1 ± 0.6% following ATRA-NC treatment, while the S-phase fraction increased from 63.6 ± 0.9% to 74.9 ± 1.2% (*p* < 0.001).

The increased representation of cells in the S phase indicates altered cell-cycle progression following treatment. Rather than describing this finding as definitive “S-phase arrest,” the present data are more appropriately interpreted as S-phase accumulation/altered cell-cycle distribution because the experiment measures phase distribution and does not directly establish the molecular checkpoint responsible for the observed change.

The observed alteration in cell-cycle distribution is consistent with the broader growth-inhibitory effects of ATRA and ATRA-NCs and complements the reduction in viability and colony-forming capacity observed in the other assays.

#### 3.3.6. Colony Formation

Colony-formation analysis demonstrated a concentration-dependent reduction in clonogenic capacity following treatment with ATRA and ATRA-NCs (Figure 9). Untreated cells formed numerous colonies, whereas both treatments reduced colony formation, with a greater reduction observed following ATRA-NC treatment at corresponding concentrations.

The quantitative analysis therefore indicates that ATRA-NCs exerted a stronger inhibitory effect on the long-term proliferative/clonogenic capacity of the treated cell population. Clonogenic assays are commonly used to assess the ability of cancer cells to retain sustained proliferative capacity and are frequently incorporated into investigations of CSC-associated phenotypes [27,29].

The greater suppression of colony formation by ATRA-NCs complements the short-term cytotoxicity results and suggests that the enhanced in vitro activity of the formulation extends to a longer-term proliferative phenotype. In the context of the present CSC-focused study, this finding is particularly relevant when considered together with the subsequent spheroid and stemness-associated gene-expression results. However, colony formation alone does not establish definitive CSC elimination or tumor-initiating capacity.

#### 3.3.7. Spheroid Formation

The effects of ATRA and ATRA-NCs on spheroid-forming capacity were evaluated as an additional functional assessment of CSC-associated properties in IMR-32 cells (Figure 10). Untreated cells formed well-defined spheroid structures, whereas treatment with ATRA reduced spheroid formation and/or size in a concentration-dependent manner. ATRA-NCs produced a greater reduction in spheroid formation at corresponding concentrations.

Spheroid formation is commonly used as an in vitro functional assay to investigate self-renewal-associated and stem-like characteristics in cancer cell populations, including neuroblastoma [27,29]. The greater inhibition produced by ATRA-NCs therefore complements the reduction in colony formation and supports an effect on CSC-associated functional properties.

Together, the colony- and spheroid-formation findings indicate that the enhanced in vitro activity of ATRA-NCs was not limited to short-term metabolic viability but was also associated with suppression of functional properties related to sustained growth and spheroid-forming capacity.

#### 3.3.8. Cell Migration

The effects of ATRA and ATRA-NCs on the migratory behavior of IMR-32 neuroblastoma cells were evaluated using a scratch-wound assay (Figure 11). Untreated cells progressively migrated into the wounded area during the 48 h observation period, whereas treatment with either ATRA or ATRA-NCs reduced wound closure in a concentration-dependent manner.

ATRA-NCs produced a greater reduction in wound closure than ATRA at corresponding concentrations. The quantitative findings therefore indicate a stronger inhibitory effect of ATRA-NCs on wound closure under the experimental conditions.

Cell migration is an important functional property associated with invasive behavior in cancer cells and may also be related to stem-like phenotypes in neuroblastoma [27,29]. The present findings therefore provide additional functional evidence of suppression of migration-associated behavior by ATRA-NCs.

Because wound closure can be influenced by both cell migration and cell proliferation, the present assay is interpreted as demonstrating reduced wound closure/anti-migratory activity under the experimental conditions, rather than definitive inhibition of metastatic potential.

#### 3.3.9. Modulation of Stemness-Associated Gene Expression

To further examine the molecular effects of ATRA and ATRA-NCs on CSC-associated characteristics, the expression of SOX2, NANOG, LGR5, OCT4, BMI-1, and ALDH1A1 was evaluated by quantitative RT-PCR (Figure 12).

Treatment with ATRA produced concentration-dependent modulation of the analyzed stemness-associated genes, while ATRA-NCs produced a more pronounced modulation at corresponding concentrations. The observed changes were consistent with the functional findings from the colony- and spheroid-formation assays.

SOX2, NANOG, and OCT4 are transcriptional regulators associated with maintenance of stem-like states, whereas BMI-1 has been implicated in self-renewal and maintenance of neuroblastoma CSC-associated populations. ALDH1A1 has also been investigated as a marker associated with stem/progenitor characteristics in neuroblastoma [27,29].

The concordance between modulation of stemness-associated gene expression and inhibition of colony- and spheroid-forming capacity provides molecular and functional support for an effect of ATRA-NCs on CSC-associated phenotypes. These findings should not, however, be interpreted as definitive evidence of CSC elimination, since changes in individual stemness-associated genes do not by themselves establish complete loss of CSC identity or tumor-initiating capacity.

#### 3.3.10. Integrated Discussion of the Physicochemical and Biological Findings

The present study demonstrates that formulation of ATRA as nanocrystals produced distinct nanoscale, colloidal, and solid-state characteristics and was associated with enhanced in vitro anticancer activity in the IMR-32 neuroblastoma model. The formulation exhibited a Z-average hydrodynamic diameter of 194 nm, although the relatively high PDI of 0.832 indicates a broad particle-size distribution. The strongly negative zeta potential of −60.1 mV, together with Poloxamer 407 stabilization, further defines the surface characteristics of the formulation. These properties provide a physicochemical basis for investigating how nanoscale formulation influences the biological activity of poorly water-soluble ATRA. The biological findings showed that ATRA-NCs produced greater reduction in cell viability than unformulated ATRA and were associated with increased apoptotic morphological changes and Annexin V/PI-defined apoptosis. The formulation also altered cell-cycle distribution, with increased representation of cells in the S phase. These findings indicate that the enhanced activity of ATRA-NCs involves multiple cellular responses rather than a single short-term viability effect.

Importantly, the effects extended to several CSC-associated functional and molecular phenotypes. ATRA-NCs produced greater inhibition of colony formation and spheroid formation than ATRA, reduced wound closure, and more strongly modulated the expression of several stemness-associated genes. Considered together, these findings support the interpretation that ATRA-NCs influence multiple characteristics associated with the stem-like phenotype of the IMR-32 model. This integrated evidence is more informative than relying on CD133 expression alone and provides a stronger basis for the CSC-focused objective of the study.

The greater activity observed with ATRA-NCs may be related to the physicochemical advantages associated with nanoscale formulation. Nanocrystals can increase the surface area available for dissolution and may improve the pharmaceutical performance of poorly water-soluble drugs [10,24]. However, the present study did not directly measure dissolution, cellular uptake, intracellular drug concentration, protein-corona formation, pharmacokinetics, or biodistribution. Therefore, these factors should be considered as possible contributors to the observed enhanced biological activity rather than as experimentally established mechanisms.

The strongly negative surface potential and Poloxamer 407 stabilization also provide a basis for considering the bio-nano interface. Nanoparticle surface charge and surface composition can influence interactions with biological media and cell membranes, while adsorbed proteins may further modify the effective biological identity of nanoscale particles [25]. The present findings demonstrate the physicochemical surface characteristics of ATRA-NCs, but dedicated uptake and mechanistic studies would be required to determine how these characteristics influence interaction with neuroblastoma CSCs.

Overall, the results support enhanced in vitro anticancer activity of ATRA-NCs and modulation of multiple CSC-associated phenotypes in the IMR-32 model. These findings provide a scientific basis for further investigation of ATRA nanocrystals as a formulation strategy for neuroblastoma, while the broader translational implications require validation in additional neuroblastoma models and appropriate in vivo pharmacokinetic, biodistribution, efficacy, safety, and formulation-stability studies.

### 3.4. Study Limitations and Future Perspectives

The present study has several limitations that should be considered when interpreting the findings. First, the biological evaluation was performed using the IMR-32 neuroblastoma model, with a CD133^+^ CSC-enriched population used for CSC-associated investigations. Although the findings provide complementary functional and molecular evidence, validation in additional neuroblastoma models would strengthen the generalizability of the observations. In addition, CD133 enrichment was used to obtain a CSC-enriched population; therefore, the findings should be interpreted in terms of CSC-associated phenotypes rather than definitive CSC identity or selective CSC eradication.

Second, the present investigation focused on physicochemical characterization and in vitro biological evaluation of ATRA-NCs. The formulation demonstrated distinct nanoscale and solid-state characteristics, providing a basis for further pharmaceutical evaluation of dissolution, drug-release behavior, and long-term storage stability. These parameters will be valuable for establishing formulation performance under different storage and delivery conditions. Similarly, the physicochemical characteristics of ATRA-NCs provide a foundation for further investigation of the bio–nano interface, cellular uptake, intracellular drug disposition, and molecular mechanisms associated with their enhanced biological activity against neuroblastoma CSC-associated phenotypes.

Third, the present findings establish the in vitro anticancer activity of ATRA-NCs and their effects on multiple CSC-associated functional and molecular phenotypes. Further translational evaluation through in vivo pharmacokinetic, biodistribution, antitumor efficacy, and safety studies will be important for determining systemic exposure, tissue distribution, therapeutic response, and safety characteristics of the formulation. Such studies will provide the necessary evidence to determine whether the enhanced in vitro activity observed with ATRA-NCs can be extended to an in vivo therapeutic setting.

Future studies should therefore extend the present findings through evaluation of dissolution and drug-release characteristics, storage stability, cellular uptake and intracellular disposition, and activity in additional neuroblastoma models. Mechanistic studies examining the molecular pathways associated with apoptosis, cell-cycle modulation, and CSC-associated phenotypes, together with in vivo pharmacokinetic, biodistribution, efficacy, and safety investigations, will further define the therapeutic potential of ATRA-NCs.

Accordingly, the conclusions of the present study are primarily applicable to the in vitro neuroblastoma model investigated, while broader translational implications require further validation. Nevertheless, the concordant effects observed across cytotoxicity, apoptosis, cell-cycle distribution, clonogenicity, spheroid formation, migration, and stemness-associated gene expression provide a multidimensional basis for further investigation of ATRA-NCs against neuroblastoma CSC-associated phenotypes.

## 4. Conclusions

The present study demonstrates the successful development and characterization of ATRA nanocrystals and provides evidence of their enhanced in vitro anticancer activity against neuroblastoma cells and CSC-associated phenotypes. ATRA-NCs exhibited nanoscale particle dimensions, a broad particle-size distribution, and a strongly negative zeta potential, while FTIR, PXRD, and DSC analyses supported preservation of the principal chemical characteristics of ATRA with modification of its physicochemical and solid-state properties following formulation.

Compared with unformulated ATRA, ATRA-NCs produced greater inhibition of cell viability and were associated with increased apoptotic responses and altered cell-cycle distribution. Importantly, the formulation also showed stronger suppression of colony- and spheroid-forming capacity, reduced wound closure, and greater modulation of stemness-associated gene expression. The concordance of these findings supports the potential of ATRA-NCs to enhance ATRA activity against CSC-associated functional and molecular phenotypes in the IMR-32 neuroblastoma model.

Overall, the findings establish ATRA-NCs as a promising in vitro formulation strategy for enhancing the anticancer activity of ATRA in a neuroblastoma CSC-focused setting. Further evaluation in additional neuroblastoma models and through mechanistic, formulation-performance, pharmacokinetic, biodistribution, efficacy, and safety studies will help determine the broader therapeutic potential of this approach.

## Figures and Tables

**Figure 1:**
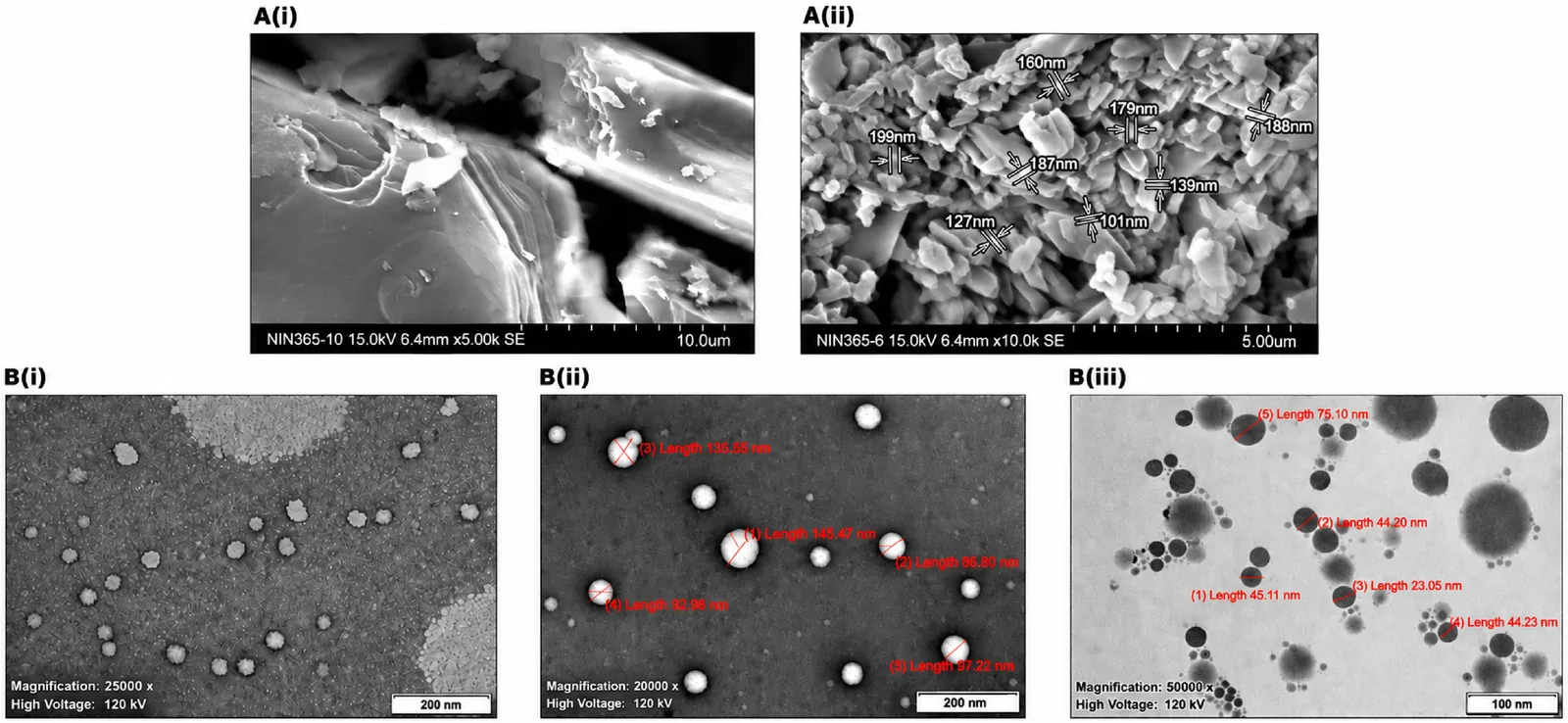
Morphological characterization of plain ATRA and ATRA nanocrystals (ATRA-NCs). (**A**) Scanning electron microscopy (SEM) images of (**i**) non-formulated plain ATRA and (**ii**) ATRA-NCs stabilized with Poloxamer 407, showing the morphological differences following nanocrystal formation. (**B**) Transmission electron microscopy (TEM) images of (**i**) non-formulated plain ATRA and (**ii**,**iii**) ATRA-NCs presented against dark and light backgrounds, respectively. Representative particle dimensions of ATRA-NCs are indicated in the corresponding micrographs. Scale bars and magnification are provided within each image.

**Figure 2:**
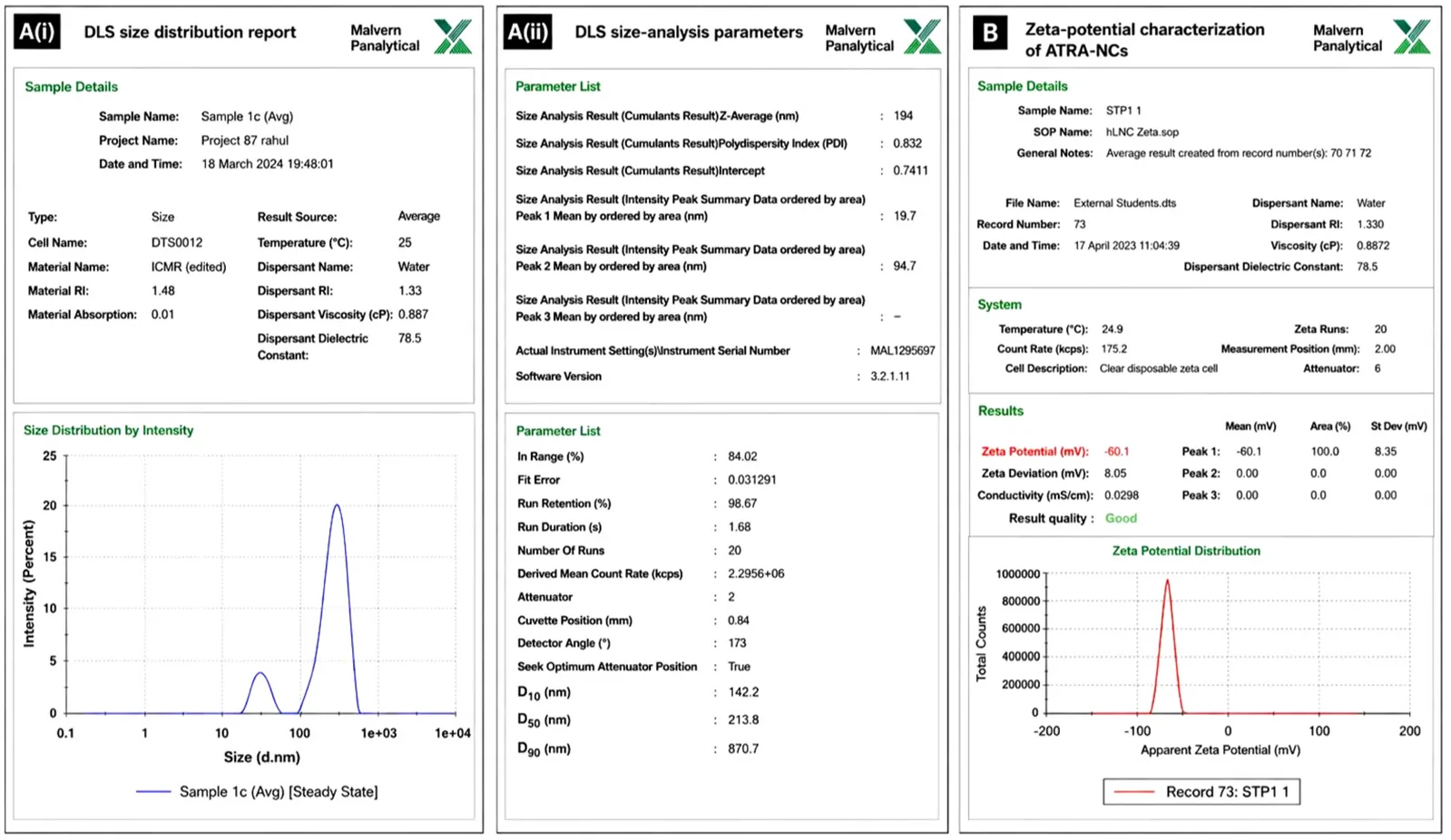
Colloidal characterization of ATRA-NCs. (**A**) Dynamic light scattering (DLS) characterization of ATRA-NCs showing (**i**) the intensity-based particle-size distribution and (**ii**) the corresponding size-analysis parameters. The formulation exhibited a Z-average hydrodynamic diameter of 194 nm and a polydispersity index (PDI) of 0.832. (**B**) Zeta potential characterization of ATRA-NCs showing a mean surface potential of −60.1 mV. The corresponding zeta potential distribution and measurement parameters are presented. Measurements were performed under the specified experimental conditions.

**Figure 3:**
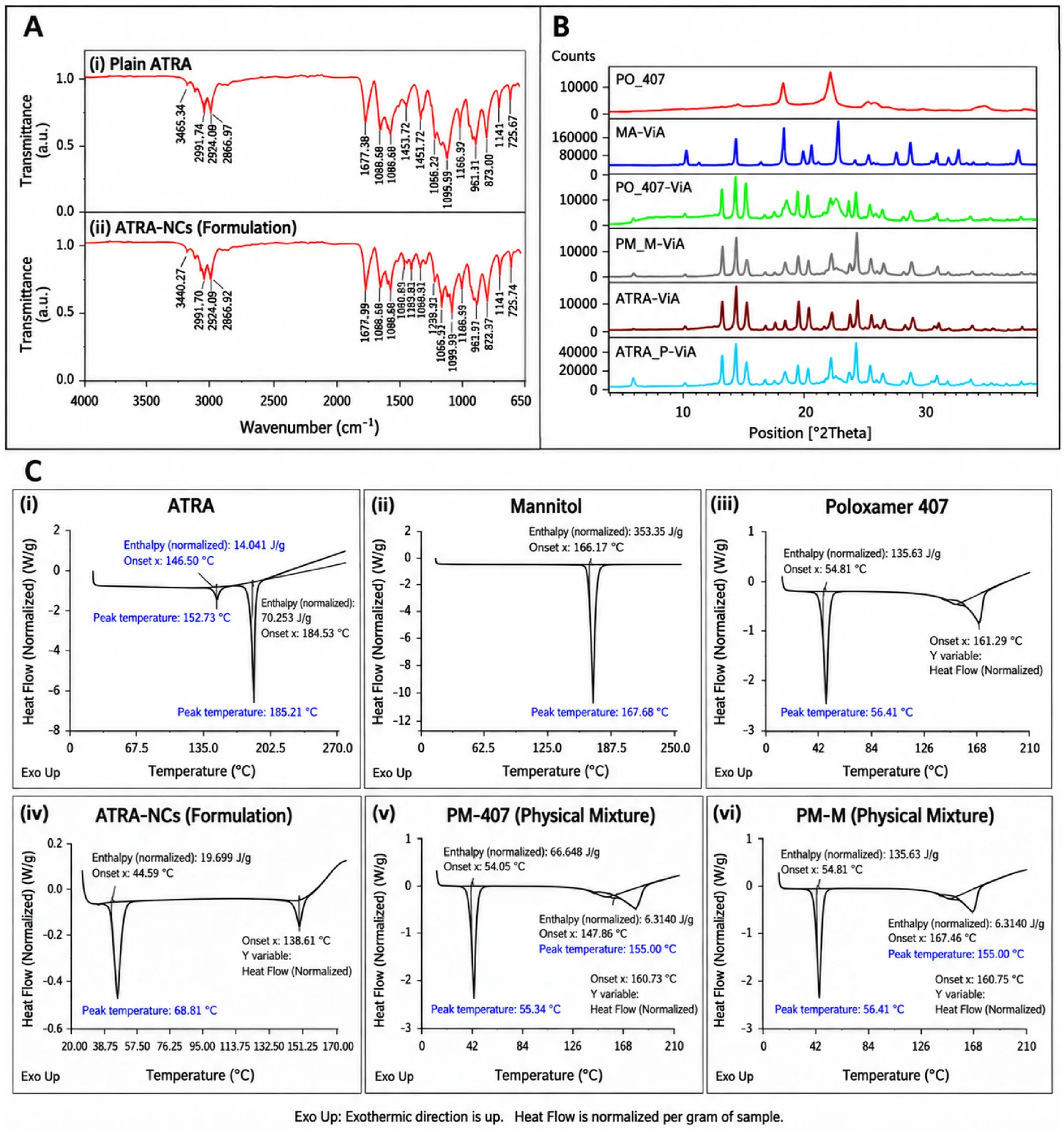
Physicochemical characterization of ATRA and ATRA-NCs. (**A**) Fourier transform infrared (FTIR) spectra of (**i**) ATRA and (**ii**) ATRA-NCs. The spectra show the characteristic vibrational bands of ATRA and the formulated nanocrystals, with the major absorption bands used to evaluate the chemical characteristics of ATRA following nanocrystal formation. Spectra were recorded over the wavenumber range of 4000–650 cm^−1^ at a resolution of 4 cm^−1^. (**B**) Powder X-ray diffraction (PXRD) analysis of ATRA-NCs and formulation components. The stacked diffraction patterns represent PO_407 (Poloxamer 407), MA-VitA (mannitol–plain ATRA), PO_407-VitA (Poloxamer 407–plain ATRA), PM_M-VitA (physical mixture of mannitol and plain ATRA), ATRA-VitA (plain ATRA), and ATRA_F-VitA (ATRA formulation). The PXRD profiles demonstrate the characteristic diffraction features of the individual components, physical mixtures, plain ATRA, and ATRA formulation. Diffraction intensity is presented as counts as a function of diffraction angle (2θ). (**C**) Differential scanning calorimetry (DSC) thermograms of ATRA, formulation components, ATRA-NCs, and physical mixtures. DSC profiles of (**i**) plain ATRA, (**ii**) mannitol, (**iii**) Poloxamer 407, (**iv**) ATRA-NCs, (**v**) PM-407 (physical mixture), and (**vi**) PM-M (physical mixture). The thermograms show the characteristic thermal transitions of the individual components and their corresponding formulations/physical mixtures. Peak temperatures and associated thermal parameters are indicated on the respective thermograms. “Exo Up” denotes the exothermic direction, and heat flow is expressed as normalized heat flow per gram of sample.

**Figure 4:**
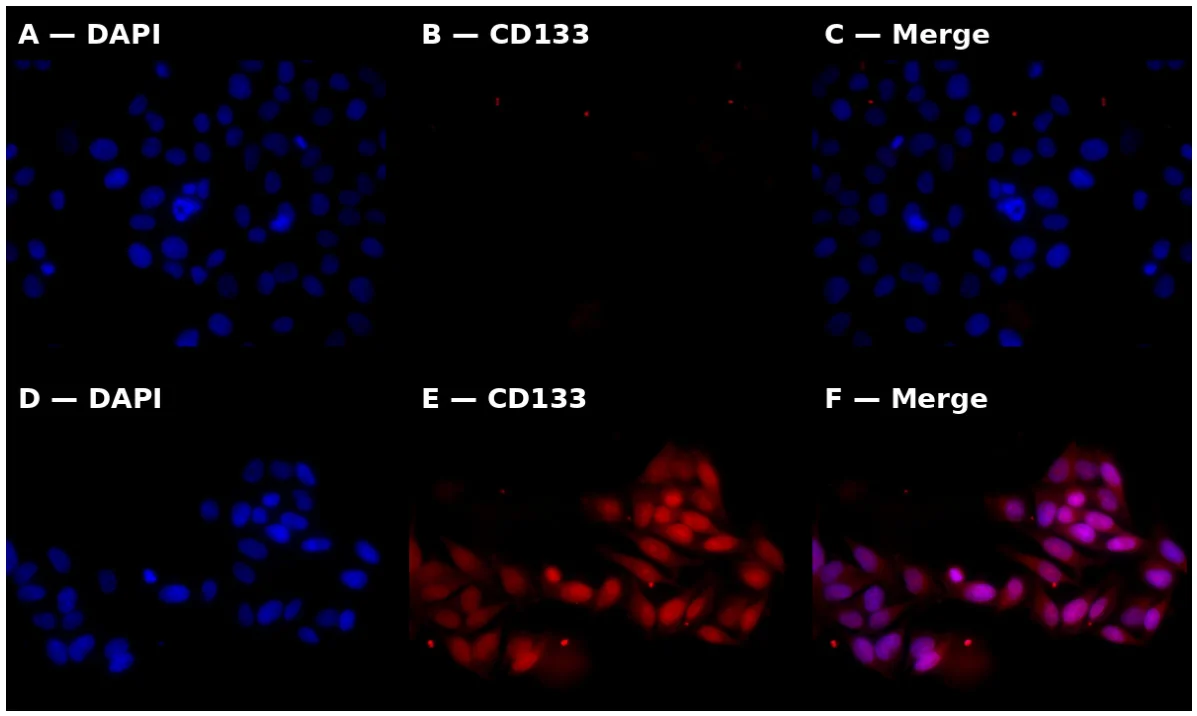
Identification and immunofluorescence characterization of CD133-positive neuroblastoma cancer stem cells (CSCs). Representative immunofluorescence images of IMR-32 cells showing DAPI-stained nuclei (blue), CD133-associated fluorescence (red), and merged images. (**A**–**C**) Representative images of the CD133-negative population, showing DAPI staining (**A**), minimal/absent CD133-associated fluorescence (**B**), and the corresponding merged image (**C**). (**D**–**F**) Representative images of the CD133-positive population, showing DAPI staining (**D**), CD133-associated fluorescence (**E**), and the corresponding merged image (**F**). The presence of CD133-associated fluorescence in the CD133-positive population supports the identification of a CD133-positive CSC-enriched population within the IMR-32 model. In the merged images, purple coloration represents the overlap of the blue DAPI nuclear signal and red CD133-associated fluorescence. Images were acquired at 40× magnification; scale bar = 20 µm.

**Figure 5:**
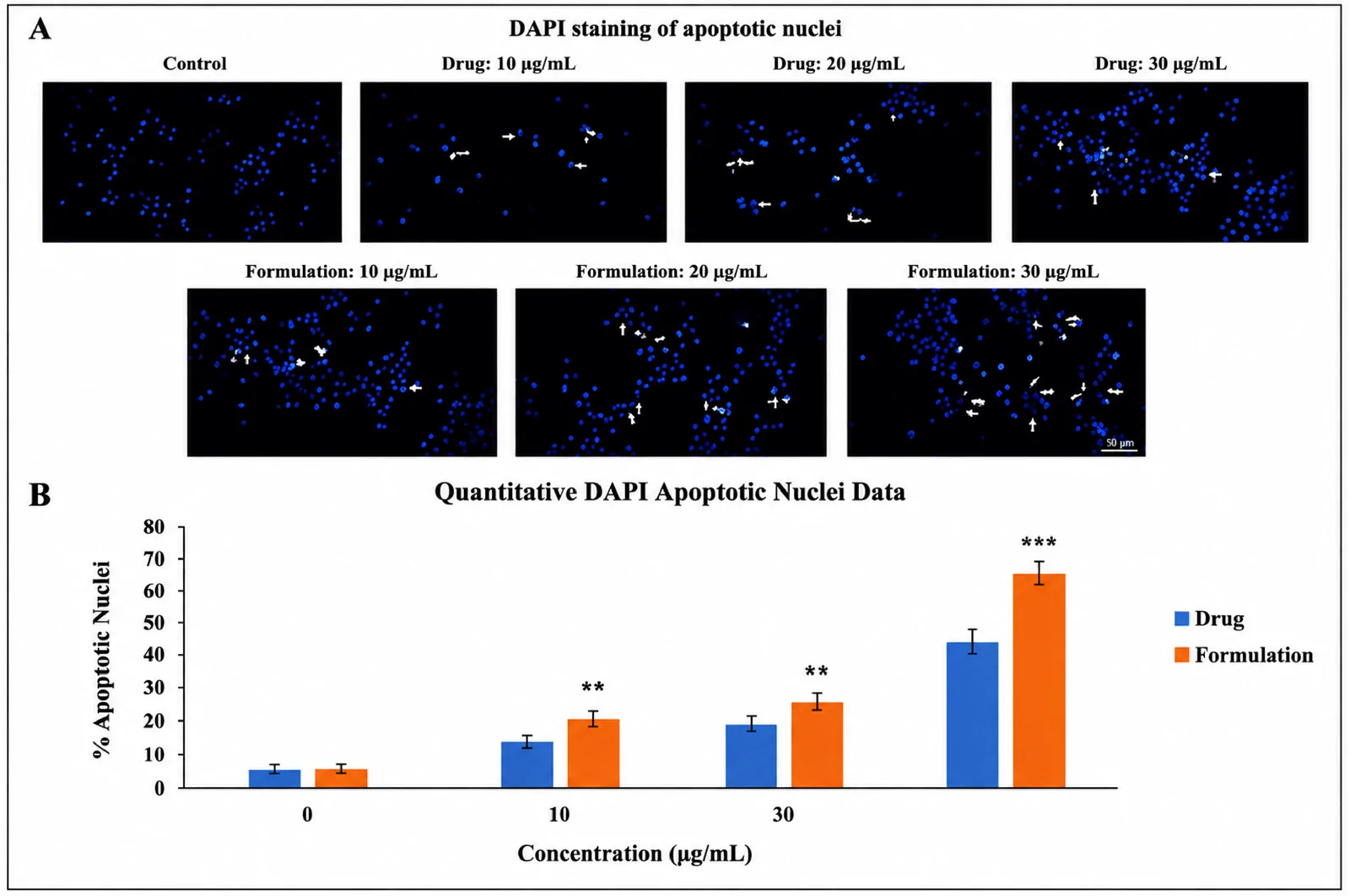
DAPI staining analysis of nuclear morphology and apoptotic changes in IMR-32 neuroblastoma cells following treatment with ATRA and ATRA-NCs. (**A**) Representative fluorescence micrographs showing DAPI-stained nuclei (blue) in untreated control cells and cells treated with ATRA or ATRA-NCs at 10, 20, and 30 µg/mL. White arrows indicate representative nuclear condensation and fragmentation. Images were acquired at 20× magnification; scale bar = 50 µm. (**B**) Quantitative analysis of apoptotic nuclei expressed as a percentage of total nuclei. Data are presented as mean ± SD (*n* = 3). Statistical significance between treatment groups was determined by one-way ANOVA followed by Tukey’s post hoc test; ** *p* < 0.01, *** *p* < 0.001.

**Figure 6:**
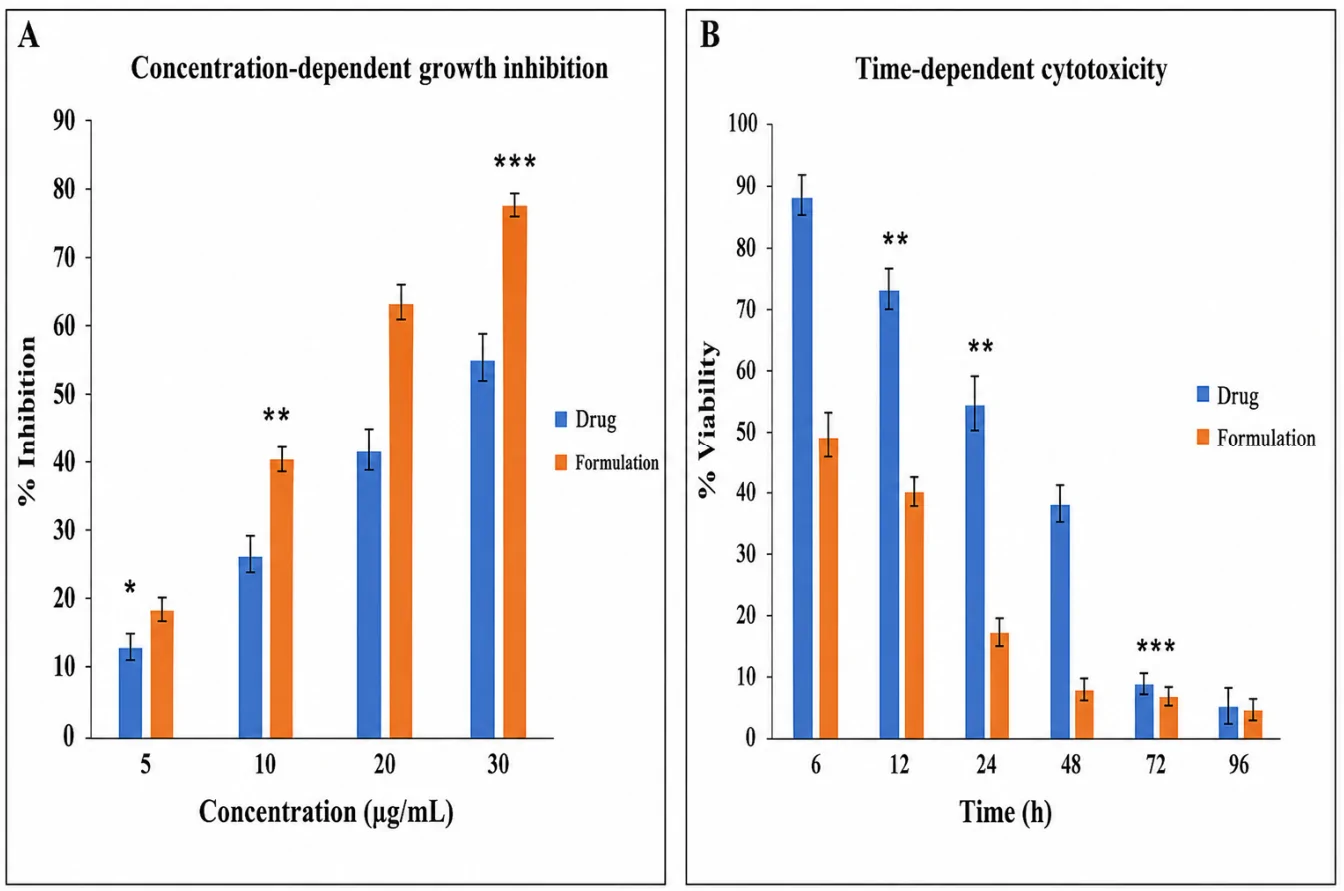
Cytotoxic evaluation of ATRA and ATRA-NCs in IMR-32 neuroblastoma cells. (**A**) Dose-dependent reduction in cell viability after 24 h treatment with ATRA or ATRA-NCs over a concentration range of 1–100 µg/mL, determined by the MTT assay. (**B**) Time-dependent cytotoxic response following treatment at the respective IC_50_ concentrations for 6–96 h. Data are expressed as mean ± SD (*n* = 3). Statistical significance was assessed using one-way ANOVA followed by Tukey’s post hoc test. * *p* < 0.05, ** *p* < 0.01, and *** *p* < 0.001 indicate significant differences between ATRA-NC- and ATRA-treated groups.

**Figure 7:**
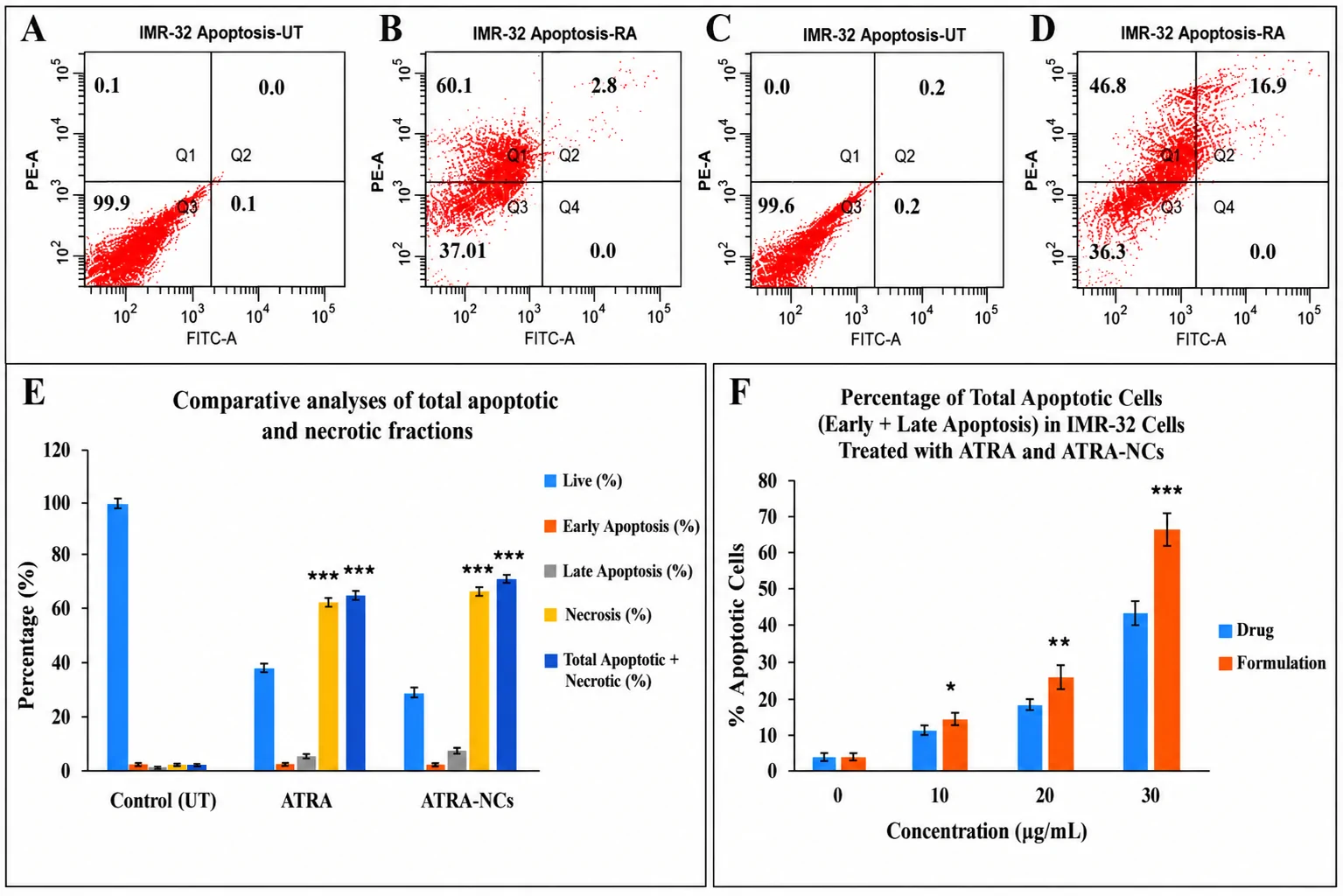
Apoptotic and necrotic responses induced by ATRA and ATRA-NCs in IMR-32 neuroblastoma cells and CSCs. (**A**–**D**) Representative Annexin V–FITC/PI flow-cytometry plots showing viable, early apoptotic, late apoptotic, and necrotic cell populations following treatment with ATRA and ATRA-NCs. (**E**,**F**) Quantitative comparison of apoptotic and necrotic cell fractions and total apoptotic populations among untreated, ATRA-treated, and ATRA-NC-treated groups. Data are presented as mean ± SD (*n* = 3). Statistical significance was analyzed using one-way ANOVA followed by Tukey’s post hoc test. * *p* < 0.05, ** *p* < 0.01, and *** *p* < 0.001. UT, untreated; ATRA, all-trans retinoic acid; ATRA-NCs, ATRA nanocrystals.

**Figure 8:**
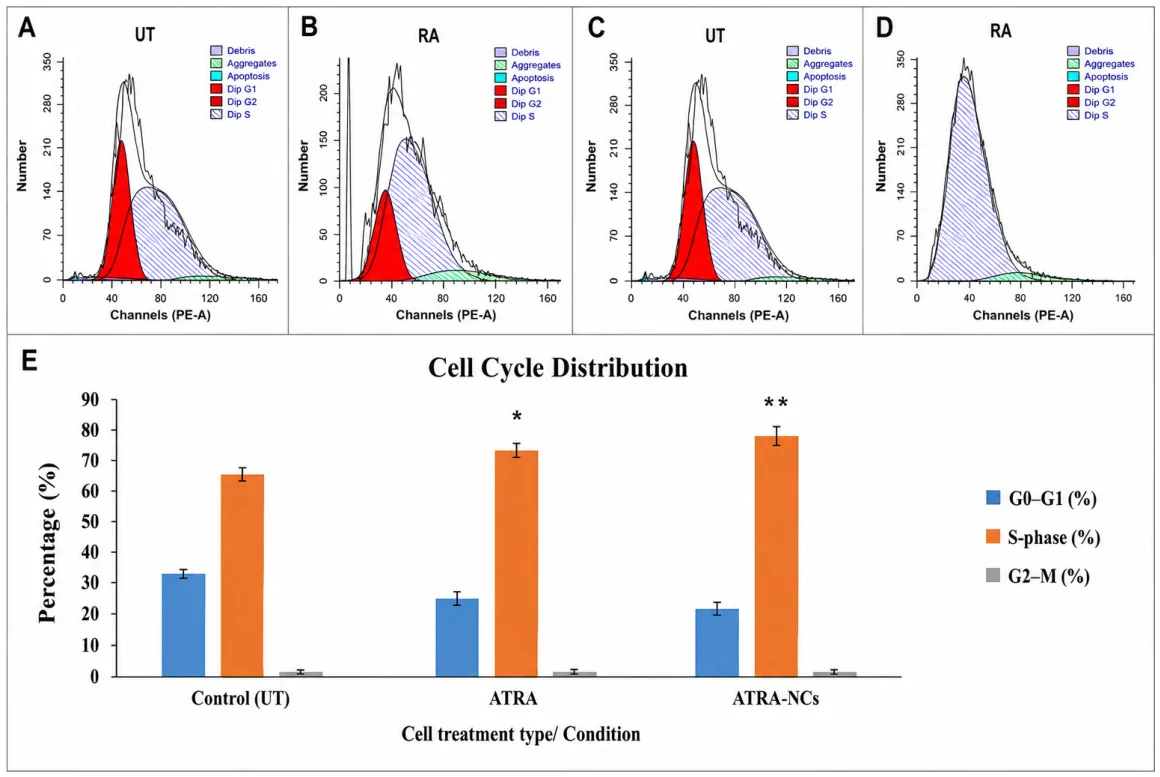
Cell-cycle distribution of IMR-32 neuroblastoma cells following treatment with ATRA and ATRA-NCs. (**A**–**D**) Representative flow-cytometry histograms showing the distribution of cells across the G0/G1, S, and G2/M phases under untreated and treatment conditions. (**E**) Quantitative comparison of cell-cycle phase distribution among untreated, ATRA-treated, and ATRA-NC-treated groups. Data are presented as mean ± SD (n = 3). Statistical significance was analyzed using one-way ANOVA followed by Tukey’s post hoc test. * *p* < 0.05 and ** *p* < 0.01.

**Figure 9:**
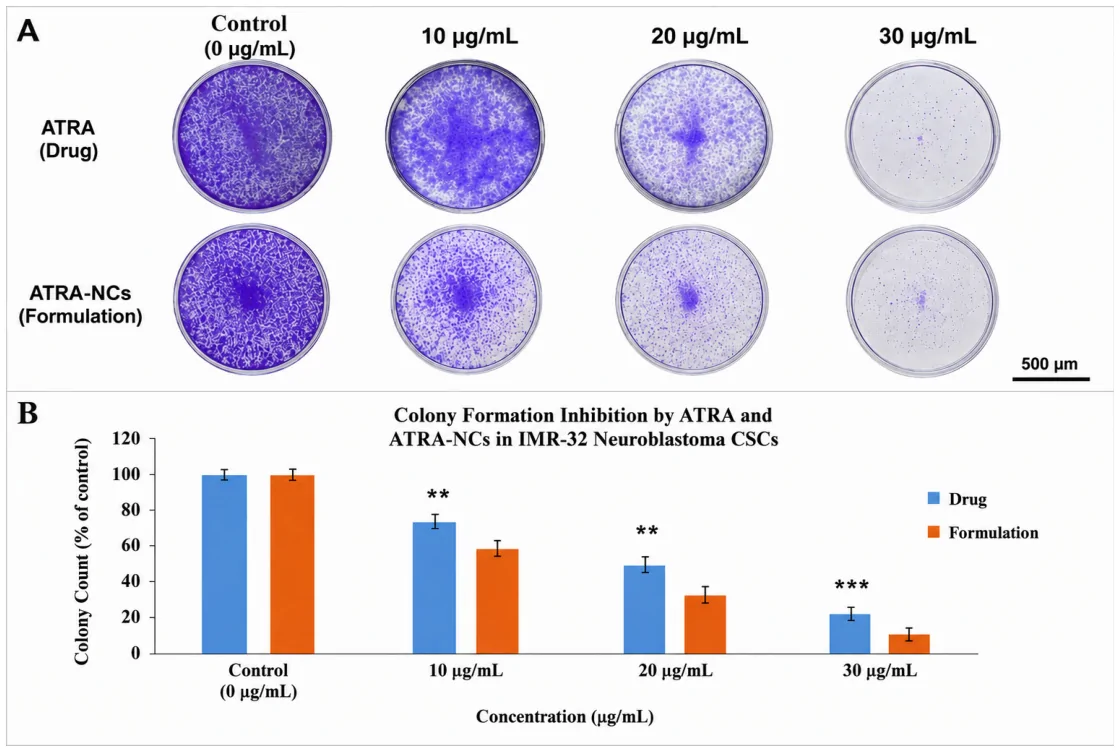
Effects of ATRA and ATRA-NCs on colony-forming capacity of IMR-32 neuroblastoma cells. (**A**) Representative images of colonies formed following treatment with ATRA or ATRA-NCs at increasing concentrations (0, 10, 20, and 30 µg/mL). (**B**) Quantitative analysis of colony formation expressed as a percentage of the untreated control. ATRA-NCs produced a greater concentration-dependent reduction in colony formation than unformulated ATRA. Scale bar = 500 µm. Data are presented as mean ± SD (n = 3). Statistical significance was analyzed using one-way ANOVA followed by Tukey’s post hoc test; ** *p* < 0.01 and *** *p* < 0.001.

**Figure 10:**
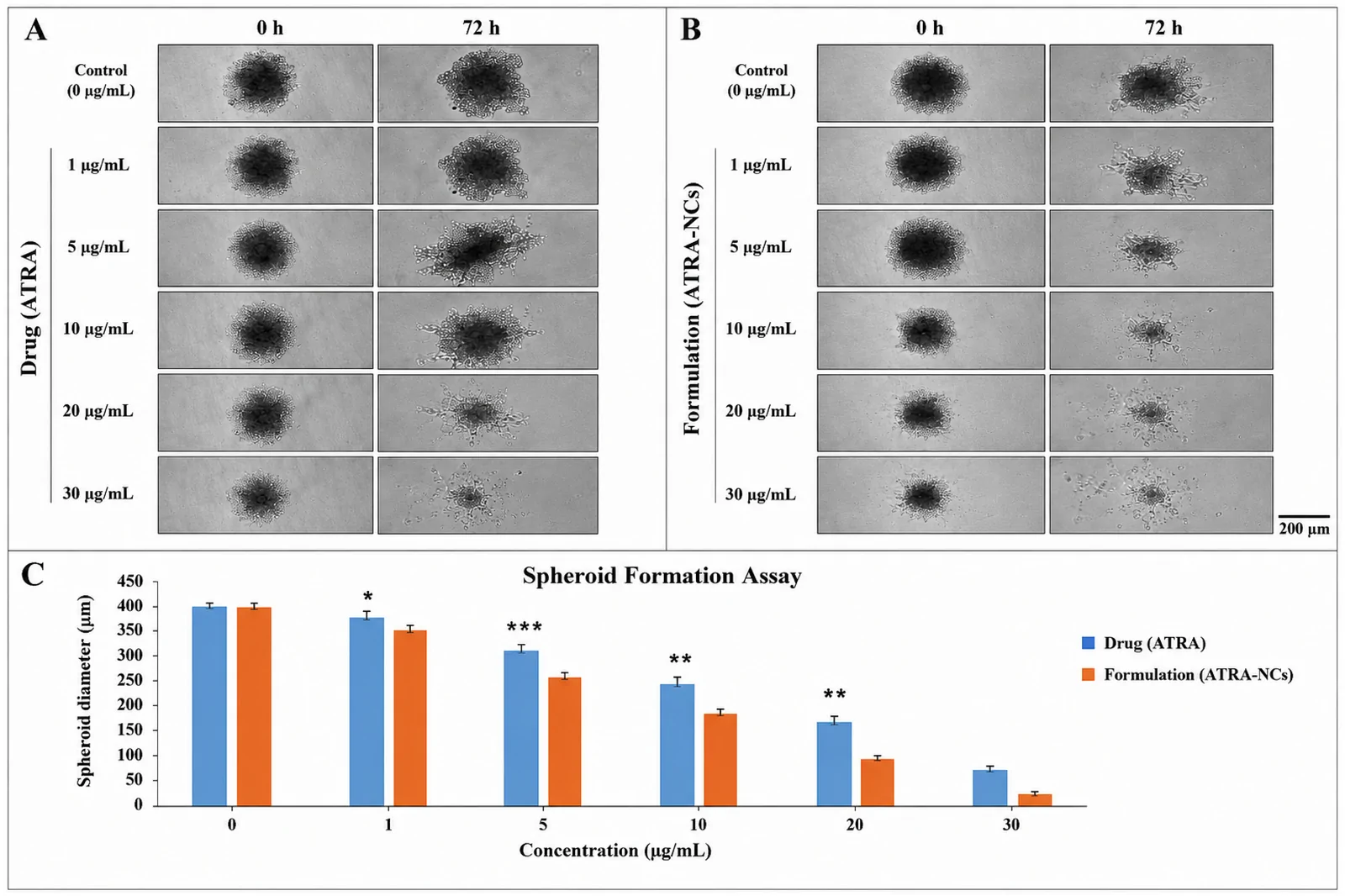
Effects of ATRA and ATRA-NCs on spheroid formation by IMR-32 neuroblastoma CSCs. (**A**) Representative spheroid images following treatment with increasing concentrations of Drug (ATRA) at 0 and 72 h. (**B**) Representative spheroid images following treatment with increasing concentrations of Formulation (ATRA-NCs) at 0 and 72 h. (**C**) Quantitative analysis of spheroid diameter following treatment with Drug (ATRA) and Formulation (ATRA-NCs). ATRA-NCs produced a greater concentration-dependent reduction in spheroid diameter compared with unformulated ATRA. Images were acquired at 10× magnification; scale bar = 200 µm. Data are presented as mean ± SD (*n* = 3). * *p* < 0.05, ** *p* < 0.01 and *** *p* < 0.001.

**Figure 11:**
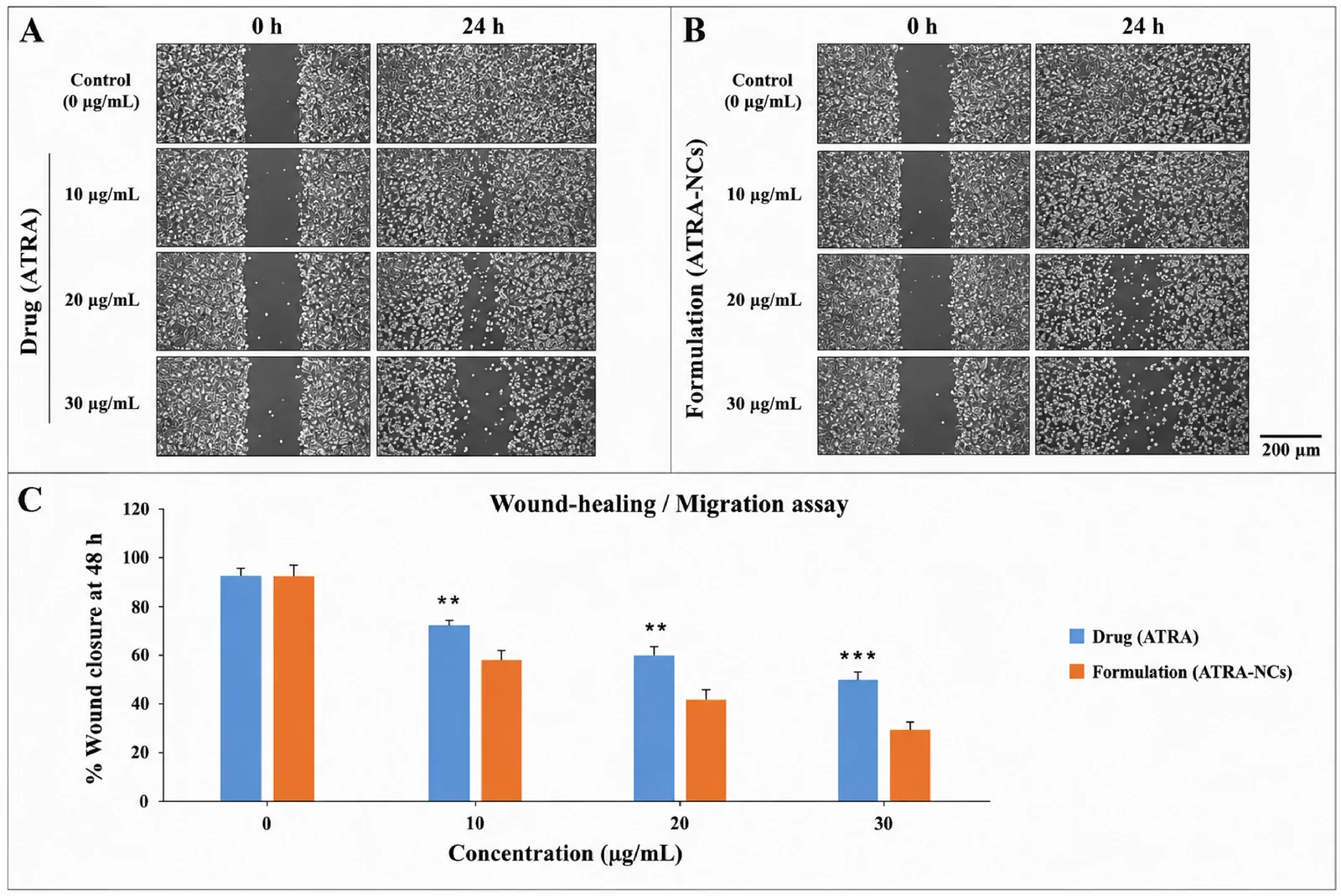
Anti-migratory effects of ATRA and ATRA-NCs in IMR-32 neuroblastoma cells. (**A**) Representative wound-healing images at 0 and 24 h following treatment with Drug (ATRA) at increasing concentrations. (**B**) Representative wound-healing images at 0 and 24 h following treatment with Formulation (ATRA-NCs) at increasing concentrations. (**C**) Quantitative analysis of wound closure (%) following treatment with Drug (ATRA) or Formulation (ATRA-NCs) at 0–30 µg/mL for 48 h. Both treatments reduced wound closure in a concentration-dependent manner, with a greater reduction observed following Formulation treatment at corresponding concentrations. Images were captured at 10× magnification; scale bar = 200 µm. Data are presented as mean ± SD (*n* = 3). ** *p* < 0.01, and *** *p* < 0.001.

**Figure 12:**
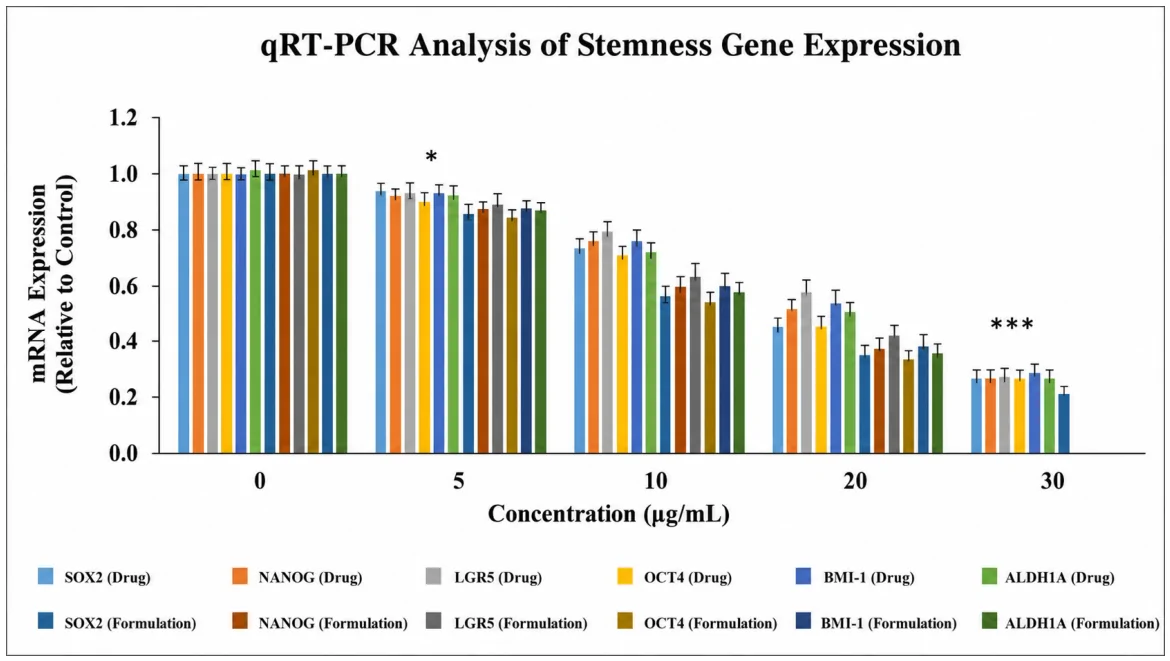
Dose-dependent modulation of stemness-associated gene expression in IMR-32 neuroblastoma CSCs following ATRA and ATRA-NC treatment. Quantitative RT-PCR analysis showing the relative expression of SOX2, NANOG, LGR5, OCT4, BMI-1, and ALDH1A1 following treatment with increasing concentrations of ATRA or ATRA-NCs. Gene expression in untreated cells was normalized to 1.0 and used as the reference. Data are expressed as mean ± SD (*n* = 3). Statistical significance was analyzed using one-way ANOVA followed by Tukey’s post hoc test. * *p* < 0.05, and *** *p* < 0.001.

**Table 1:** Apoptosis analysis of IMR-32 cancer cells and CSCs following treatment with ATRA and ATRA-NCs.

S. No.	Cell Type	Treatment	Live Cells (%)	Early Apoptosis (%)	Late Apoptosis (%)	Necrosis (%)	*p*-Value	S. No.
1	IMR-32 cancer cells	UT	99.9 ± 0.2	0.1 ± 0.0	0.0 ± 0.0	0.1 ± 0.0	—	1
2	IMR-32 cancer cells	ATRA	37.0 ± 1.5	0.0 ± 0.0	2.8 ± 0.4	60.1 ± 2.1	<0.01	2
3	IMR-32 CSCs	UT	99.6 ± 0.3	0.2 ± 0.0	0.2 ± 0.0	0.0 ± 0.0	—	3
4	IMR-32 CSCs	ATRA-NCs	36.3 ± 1.1	0.0 ± 0.0	16.9 ± 1.2	46.8 ± 1.5	<0.001	4

Data are presented as mean ± SD from three independent experiments (*n* = 3). Statistical significance was analyzed using one-way ANOVA followed by Tukey’s post hoc test.

**Table 2:** Cell-cycle phase distribution in IMR-32 cancer cells and CSCs following treatment with ATRA and ATRA-NCs.

S. No.	Cell Type	Treatment	G0/G1 (%)	S Phase (%)	G2/M (%)	*p*-Value	S. No.	Cell Type
1	IMR-32 cancer cells	UT	33.6 ± 0.9	66.4 ± 1.1	0.0 ± 0.0	—	1	IMR-32 cancer cells
2	IMR-32 cancer cells	ATRA	25.4 ± 0.7	74.6 ± 1.3	0.0 ± 0.0	<0.01	2	IMR-32 cancer cells
3	IMR-32 CSCs	UT	36.4 ± 0.8	63.6 ± 0.9	0.0 ± 0.0	—	3	IMR-32 CSCs
4	IMR-32 CSCs	ATRA-NCs	25.1 ± 0.6	74.9 ± 1.2	0.0 ± 0.0	<0.001	4	IMR-32 CSCs

## Data Availability

All data supporting the findings of this study are included in the article and its accompanying figures and tables, further inquiries can be directed to the corresponding author.
